# Malaria and HIV/AIDS Coinfection in Patients Under Highly Active Antiretroviral Therapy at the Regional Hospital of Bafoussam (West Cameroon)

**DOI:** 10.1155/2024/5520975

**Published:** 2024-10-28

**Authors:** Romeo Tankoua-Tchounda, Jacques Nack, Christian Mbohou Nchetnkou, Desire Leonard Keptcheu Tchankwe, Michel Lontsi-Demano, Estelle Essangui, Alex Kevin Tako Djimefo, Leopold Gustave Lehman

**Affiliations:** ^1^Department of Biology of Animal Organisms, Faculty of Sciences, University of Douala PO 24157, Douala, Cameroon; ^2^Department of Animal Biology, Faculty of Sciences, University of Dschang, PO Box 067, Dschang, Cameroon

**Keywords:** AIDS, Cameroon, HIV-malaria coinfection, HIV-positive, viral load

## Abstract

**Background:** Malaria and HIV/AIDS are the two most common infections responsible for morbidity and mortality in sub-Saharan Africa. The studies were carried out worldwide. However, no study has targeted HIV-positive patients at the Bafoussam Regional Hospital (West Cameroon), one approved treatment center, where patients are adhering well to their HIV treatment. The objective of this study was to identify the *Plasmodium* species and to determine the prevalence of the malaria parasite in relationship with associated factors in HIV+ patients followed at the Bafoussam Regional Hospital.

**Methods:** A prospective study included 585 patients who responded to the questionnaires from May to December 2021. Parents or legal guardians of children under 15 responded on their behalf on knowledge, attitudes, and practices towards malaria. Venous blood samples collected in EDTA tubes were subjected to malaria diagnosis by rapid tests (Standard Diagnostics Boline), and the results were confirmed by microscopy. The blood count was undertaken on hematology analyzer (Mindray Company, Shenzhen, China).

**Results:** *Plasmodium vivax* (4.3%) and mostly *Plasmodium falciparum* (95.7%) were identified. In this study population, 46 (7.9%) of the patients carried one or the other *Plasmodium* species, and 532 (90.9%) had undetectable HIV viral loads. The prevalence of malaria was significantly higher among those using traditional pharmacopoeia (9 (16.7%)) compared to patients taking generic treatments (37 (7.0%)) (*p* < 0.01; OR: 2.69). Factors associated with malaria prevalence, such as sociodemographic characteristics, viral load, type of protocol, duration of antiretroviral treatment, monthly income, subdivision, and knowledge attitudes and practices towards malaria, showed no significant differences (*p* > 0.05).

**Conclusion:** This study showed that HIV+ patients were carriers of *Plasmodium falciparum* and *Plasmodium vivax* with an appreciable overall prevalence. The only factor influencing the prevalence of malaria was using traditional medicine.

## 1. Introduction

Malaria and HIV/AIDS are two ongoing pandemics in sub-Saharan Africa, Southeast Asia, and South America. These conditions pose enormous global health challenges, each causing more than three million deaths in 2007 and affecting millions of people each year [1, 2]. In 2020, an estimated 37.6 million people were living with human immunodeficiency virus (HIV), and 1.5 million people were newly infected [3]. In the Central African subregion, Cameroon is the 2nd country, after Nigeria, where the burden of the HIV pandemic is considerable with approximately 500,000 infected people [4, 5]. According to the WHO's malaria report, there are 241 million cases and 627,000 deaths in 2020. However, the greatest burden was in sub-Saharan African countries, which accounted for 95% of all morbidity and mortality [6]. Cameroon is the 11^th^ country with a large malaria burden in the world with a prevalence of 2.9%, 3rd in the Central African subregion [6], and is endemic in all 10 regions of the country [7]. This makes the condition one of the leading causes of morbidity among HIV-infected individuals in sub-Saharan Africa [8].

People living in malaria-endemic areas and considered semi-immune to malaria are still more likely to develop clinical malaria if they are infected with HIV [9]. Indeed, repeated exposure to *Plasmodium falciparum* in areas of stable transmission progressively induces the constitution of a complex natural state of premunition [10]. Studies have shown that malaria infection increases plasma HIV viral load, even in asymptomatic parasitemia [11, 12]. This increase in HIV plasma viral load during the malaria episode is in the absence of treatment [13].

HIV infection increases the incidence of malaria attacks the more profound the immunosuppression, but the severity and mortality of attacks are only increased in areas of unstable malaria [14, 15]. Globally, the risk of uncomplicated and severe malaria episodes is higher in HIV-positive patients coinfected with malaria, especially in at-risk groups such as pregnant women and children [9, 16]. This HIV-malaria coinfection is particularly serious for pregnant women in whom complications such as life-threatening anemia and placental malaria infection are common [17]. These cases of malaria are treated with *generic medicines*. The studies in sub-Saharan Africa have found that HIV-infected patients use *herbal treatments* for malaria and other conditions while on antiretroviral therapy [18]. In 2023, the WHO is calling for plant-based treatment to be integrated into disease management [19].

Worldwide, a minority of studies have evaluated the undetectable viral load of HIV-positive patients in recent years. In Cameroon, there is little data on malaria in patients living with HIV in the hypoendemic zone and patients followed at the Bafoussam Regional Hospital have not been the subject of a study to evaluate either the prevalence or the parasite density, or the relationship between the prevalence of malaria and HIV viral load. The objective of this epidemiological study presenting the current situation of HIV/malaria coinfection was to identify the *Plasmodium* species present and to determine the prevalence of malaria in relation to associated factors in HIV+ patients followed at the Bafoussam Regional Hospital.

## 2. Materials and Methods

### 2.1. Study Site

**Figure 1:**
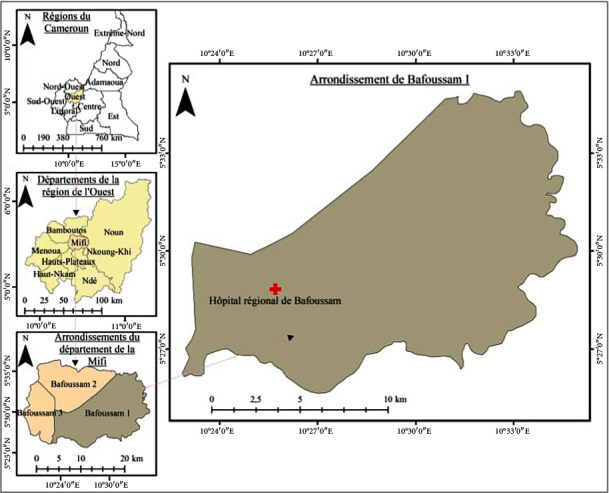
Geographic location of the Bafoussam Regional Hospital, Mifi Division, West Region of Cameroon.

The present study was conducted at the Bafoussam Regional Hospital, located in the Mifi division, West Region of Cameroon (Figure 1). This hospital is the referral and care center for people living with HIV in the region. HIV-positive patients come from several divisions in the region and from other parts of the country.

The West Region is made up of eight divisions covering an area of 13,892 km^2^ with over 100 inhabitants per km^2^. The Mifi division has Bafoussam as its capital. It has approximately 301,456 inhabitants [20]. It is bordered to the north by the Bamboutos division, to the south by the Haut-Plateaux and Koung-Khi subdivisions, to the east by the Noun division, and to the west by the Menoua subdivision (Figure 1). These divisions are populated with an estimated 292,410, 455,083, 80,678, and 372,244 inhabitants, respectively, in Bamboutos, Noun, Haut-Plateaux, and Menoua [20].

The study area is characterized by an equatorial, high–altitude monsoon climate with four types of seasons, namely, the long dry season (November–mid-March), the short rainy season (mid-March–May), the short dry season (June–July), and the long rainy season (August–October) [21]. It is located 5°–7° N latitude and 8°–20° E longitude. The temperature varies from 16°C to 27°C. Rainfall averages 2000 mm per year, spread over the period from March to November [22]. Its average altitude is 1450 m. These climatic characteristics as well as seasonal variations create favorable conditions for the maintenance and development of mosquitoes [23, 24].

### 2.2. Data Collection

Questionnaire sheets were used to collect sociodemographic information. These included age, gender, place of residence, education level, marital status, occupation, and knowledge on malaria. Data on viral loads and types of antiretroviral treatments were collected from the file of each HIV-positive patient at the hospital's HIV treatment center.

### 2.3. Laboratory Analysis Procedure

Parasitological data were obtained by microscopic observation while hematological parameters were obtained using the BC 2800 (Mindray Company, Shenzhen, China). After obtaining participants' consent, 5 mL of blood was collected by venipuncture at the elbow with needles (vacutenair) adapted to the vacutenair holder and stored in ethylene diamine tetra acetate (EDTA) tubes. Once in the laboratory, these blood samples were used for rapid diagnostic tests (RDTs), microscopic analysis (blood smears), and blood counts.

To screen for malaria parasites, we used malaria RDT kits as described by the manufacturer (SD Standard Diagnostics, Inc) (Standard Diagnostics Boline, 2016). All test devices were allowed to stand at room temperature for 15 min. Five microliters of EDTA whole blood sample was added to the sample test device, followed by three drops of cleaning buffer. The reaction was left at room temperature for 15 min. The appearance of a distinct red line at the control and test areas was observed for interpretation of the result according to the manufacturer's instructions. Thick and thin smears were immediately prepared for each blood sample collected. The slides were allowed to air dry. After drying, the thin smears were fixed with absolute methanol very carefully so that they did not touch the thick smear. The thin smear was allowed to air dry (fixation). The thick and thin smears were then stained with 10% Giemsa for 10 min. After this, the stain was removed by rinsing with tap water and then allowed to air dry on a drying rack. When the blood smears were completely dry, a drop of immersion oil was placed on a suitably stained area and placed under the 100x objective of a binocular microscope with built-in illumination for observation of malaria parasites [25]. Parasite density was calculated while using the white blood cell made with the Mindray hematology analyzer [25].

The total WBC counted is the number of white blood cells given by the Mindray hematology analyzer (blood count). Number of white blood cells counted simultaneously is the number of white blood cells counted on the smear.

 $$\begin{array}{c} \text{Number of parasites per }\mu \text{L}=\frac{\text{Number of parasites counted }}{\text{Number of WBCs counted simultaneously}}\times \text{Total WBCs counted} \end{array}$$

### 2.4. Statistical Analysis

The data collected were recorded in Microsoft Excel 2013 software and then analyzed with SPSS Version 22.0 (Statistical Package for Social Sciences) software, and the rib ratio was calculated using Medcalc Version 14.8.1. Descriptive statistics was used to highlight tables and graphs. A chi-square (*χ*^2^) test was used to compare the prevalence of malaria infection according to sociodemographic characteristics, viral load, using traditional pharmacopoeia, type of protocol, duration of antiretroviral treatment, monthly income, subdivision, and knowledge attitudes and practices towards malaria, while the odds ratio was calculated to estimate the risk attributable to the different factors with confidence intervals. *p* values less than 0.05 were considered statistically significant.

### 2.5. Ethical Considerations and Authorizations

Patients voluntarily consented to participate in the study by signing an information sheet. In the case of children and adolescents (under 20 years of age), their parents or legal guardians signed on their behalf. Ethical authorization was obtained from the Institutional Research Ethics Committee for Human Health of the University of Douala (CEI-UDo) Reference number: 2605 CEI-UDo/04/2021. Authorization for the collection of blood was obtained from the manager of the Bafoussam Regional Hospital. For reasons of confidentiality, patients were identified anonymously using identification numbers. The results of the participants with malaria were referred to the head of the confirmed treatment center (CTA).

## 3. Results

### 3.1. Sociodemographic Characteristics of the Study Population

A total of 585 patients aged 3–81 years participated in the study. The mean age of the participants was 43.48 ± 13.86 years and a median of 44.0. The female gender was more represented (73.7%) than the male gender (26.3%) with a sex ratio F/M of 2.79/1. The age group 41–50 years was the most represented (30.4%), and the least represented was under 20 years (5.6%). The marital status shows that married patients were more represented (52%) and divorced patients were less represented (5.5%). According to the level of education, participants at the high school level (47%) were more represented, and those at the university level were the least represented (7.7%) (Table 1).

### 3.2. Age Distribution and Level of Education of HIV-Positive Patients According to Gender

Of the 585 HIV–positive patients, all age groups were more represented by females than males. Patients with all levels of education were more likely to be female than male (Table 1).

### 3.3. Breakdown of Participants' Level of Education According to Age

Among these HIV-positive patients, the most illiterate were those aged 51–60 years (29.4%), and the least illiterate were those aged 20 years or less (0%) (Table 2). Participants with primary, secondary, and university education were more represented in the 41–50 age group (28.5%, 32.4%, and 31.1%), and those aged 20 and under were less represented (3.7%, 9.1%, and 0%) (Table 2).

### 3.4. HIV Viral Loads of Patients

Of the 585 patients, 21 (3.6%) had viral loads greater than 1000 copies per mL of blood, 32 (5.5%) between 50 and 1000 copies, and 532 (90.9%) less than 50 copies.

### 3.5. Malaria Prevalence

During this study, 46 patients were found to be malaria positive, representing a prevalence of 7.9%. Two plasmodium species were identified: 44 cases of *Plasmodium falciparum* (95.7%) and 2 cases of *Plasmodium vivax* (4.3%).

### 3.6. Parasite Density for Positive Cases

A majority of HIV-positive malaria–infected patients 29 (63%) had a parasite density between 401 and 8260 trophozoites/*μ*L while those with a parasite density of 84–400 were the fewest 17 (37%).

### 3.7. Distribution of *Plasmodium* Species and Density According to Sex and Age

Two (2) cases of *Plasmodium vivax* were identified in females. The age range of ≤ 20 years and 51–60 years was infected with *Plasmodium vivax* (Table 3). Parasite density by gender shows that the female gender had higher parasitemia between 84 and 400 trophozoites/L (37.5%) and 401 and 8260 trophozoites/L (93.8%) compared to the male gender's 84 and 400 (35.7%) and 401 and 8260 (64.3%). As a function of age, the 21–30 age group had a higher parasite density than the others (50%) (84–400) and the age group less than or equal to 20 years (401–8260) (100%) (Table 3).

### 3.8. Malaria Prevalence According to Subdivision

The study population consisted of patients from several divisions of the Western Region and patients from other regions. The predominant population were patients from Mifi (63%), followed by Noun (11%), Bamboutos (8%), and others (6.3%). Prevalence did not vary significantly by department (Table 4). The division with the highest malaria prevalence was Haut-Nkam, Nde, and Noun with rates of 33.3%, 18.2%, and 11.1%, respectively. The prevalence did not vary significantly between these divisions (Table 4).

### 3.9. Prevalence of Malaria According to Viral Loads

*Plasmodium* was present in all two viral load ranges in HIV-positive patients. Patients with a viral load below 50 copies per mL of blood (532 (90.9)) had a higher prevalence of infection (7.3% (39 cases)). In contrast, those with viral load levels above or equal to 50 copies/mL had a lower prevalence of infection (13.2% (7 cases)). The prevalence of malaria infection showed no significant difference according to viral loads (*p* = 0.13; OR (95%CI) = 0.52 (0.22–1.22).

### 3.10. Parasite Density of Malaria-Positive Cases According to Viral Load

Of the 46 malaria-positive patients, 39 had a viral load of less than 50 copies/mL, with 16 (41%) patients having a parasite density of between 84 and 400 trophozoites/*μ*L and 23 (59%) patients between 401 and 8260 trophozoites/*μ*L. However, 7 patients had a viral load greater than or equal to 50 copies/mL: 1 (14.3%) patient with a parasite density of between 84 and 400 trophozoites/*μ*L, and 6 (85.7%) patients with a parasite density of between 401 and 8260 trophozoites/*μ*L.

### 3.11. Antimalarial Treatment in Relation to Viral Load

In this study with a population of 585 participants, two antimalarial treatments were used. Their distribution according to viral load showed that 483 patients were using generic drugs, and 49 plants had a viral load of less than 50 copies/mL. Among those whose viral load was greater than or equal to 50 copies/mL, 48 patients used generic medicines, and 5 used plants.

### 3.12. Malaria Prevalence According to Clinical Factors and Monthly Income

Malaria infection was higher in patients on antiretroviral therapy for less or equal to 12 months (13.6%) than in those on it for more than 36 months (7.1%). This infection was higher among participants on first-line antiretroviral treatment (8.1%), and those on third-line treatment were not infected (Table 5). According to monthly income, participants with a monthly income above or equal to 35,000 CFA francs were more infected (8.6%), and the least infected were those with an income less than 35,000 CFA francs (7.4%) (Table 5). The prevalence of infection according to the duration of antiretroviral treatment, line of treatment, and monthly income showed no significant difference.

### 3.13. Prevalence of Malaria According to the Type of Antimalarial Treatment Used

The prevalence of *Plasmodium* infection in patients was significantly higher in patients usually managed for malaria using traditional medicines (9/54 (16.7%); *p* = 0.01; OR (95%CI) = 2.69 (1.21–-5,9)) compared to patients taking generic treatments (37/531 (7.0%)).

### 3.14. Malaria Prevalence According to Sociodemographic Characteristics

Male HIV–positive patients were more infected (9.1%) than female HIV–positive patients (7.4%) (Table 6). The age group above 60 years was more infected with malaria. Similarly, single patients were more infected. According to the level of education, illiterates were more infected, and the least infected were university graduates with a prevalence of 11.8% and 4.3%, respectively. The prevalence of *Plasmodium* showed no significant differences according to gender, age groups, marital status, and education level.

### 3.15. Prevalence of Malaria According to the Knowledge, Attitudes, and Practices Towards Malaria of Patients Living With HIV

*Plasmodium* infection was higher among participants who did not know whether malaria was dangerous or not for their health (8.6%) than among those who knew that malaria was not dangerous (4.2%). This infection was higher among patients who declared that the mosquito bite was the means of malaria transmission (8.5%) and absent among those who declared a lack of cleanliness and via food (0%) (Table 7). Participants with no knowledge of the type of mosquito responsible for malaria transmission were more infected with malaria (8.2%) than those with good knowledge (5.9%) (Table 8). Malaria prevalence was higher among patients who slept under long-lasting insecticidal nets (LLINs) (11.5%) compared to those who did not sleep (7.7%). Patients whose LLINs came from the Ministry of Public Health were more infected (8.8%) and absent among those who had paid for them or acquired them by other means (0%). The prevalence of infection according to knowledge, attitudes, and practices towards malaria revealed no significant differences.

### 3.16. Knowledge Attitudes and Practices Towards Malaria of Patients Living With HIV

HIV-positive patients aged 3–81 years (*n* = 585) were asked about malaria transmission and prevention. Of these participants, 81.9% reported having some knowledge about the danger that malaria can cause. The majority of respondents reported that the mosquito bite was the means of malaria transmission (74.2%), and 21% had no idea about the means of transmission (Table 8). In this population, 10.4% had knowledge of the type of mosquito responsible for malaria transmission. Overall, (69.9%) slept under LLINs. Of these, 77.9% had their LLINs from the Ministry of Public Health, and 11.1% had no LLINs. The majority of these participants (32.3%) had one to three malaria attacks per year. The most frequently mentioned prevention method was cleaning the environment (17.3%), followed by cleaning the environment and using insecticide (4.1%) and using insecticide (2.2%) (Table 8).

## 4. Discussion

The objective of this prospective study was to identify the species and determine the prevalence of malaria and the factors associated with its maintenance in HIV-positive patients at the Bafoussam Regional Hospital. The overall prevalence of malaria was 7.9%. Two plasmodial species were detected. These were *Plasmodium falciparum* and *Plasmodium vivax*. This prevalence is relatively low to that obtained in Cameroon by Eyong et al. in Bamenda (23.81%) [26] and Mekachie Sandie et al. in Limbe (14.1%) [27], Cameroon (14.1%), Kenya (64.3%) [28], and Nigeria (11.5%) [29]. The low prevalence in the present study would be explained by the reduction of the viral load made undetectable in patients, which favors the reconstitution of their immune system with the administration of antiretrovirals combined with compliance with these drugs. According to Suleiman et al. [30], the introduction of highly active antiretroviral therapy in recent years worldwide as the main treatment for HIV has led to a reduction in the frequency of infections, including those caused by enteroparasites, and has improved the clinical and laboratory results of patients. However, numerous clinical reports and laboratory results suggest that the control of opportunistic parasitic infections in HIV-positive people on HAART is also induced by anti-HIV protease inhibitors, which inhibit parasite aspartyl proteases [31]. Furthermore, Andrews et al. [32] demonstrated the activity of several protease inhibitors against *P. falciparum* in vitro and *Plasmodium chabaudi* in vivo, as well as against clinical isolates of *P. falciparum* and *Plasmodium vivax* [33]. The presence of *Plasmodium vivax* would, therefore, be related to the multiple movements of patients in high-risk areas, including travelers and foreigners. A recent study noted the existence of this species in Santchou, Dschang, and Kye'-Ossi in Cameroon [34]. This species was observed in Dschang in 2017 [35] and in the South West and South regions of Cameroon in 2014 [36]. Similar observations of HIV and *Plasmodium vivax* coinfection have been made in Nigeria [12] and Brazil [37, 38]. Work on malaria in adult patients attending the district hospitals of Bamendjou and Foumbot in the Western Region of Cameroon revealed prevalences of 47.06% and 19.8%, respectively [39]. Based on these observations, antiretroviral treatment leads to a reduction in the prevalence of malaria in HIV patients undergoing follow-up. It appears that antiretroviral treatment, by allowing immune restoration, has the capacity to reduce the risk of HIV-related malaria [40]. A study of blood donors at the Ebolawa Regional Hospital in Cameroon revealed a higher prevalence of 73.24% [41].

The prevalence of infection in patients with both species of *Plasmodium* was high in patients who usually managed their malaria with traditional medicines (*p* < 0.05). This could be attributed to noncompliance with dosage and dose during malaria treatment or to the fact that some of the treatments used are not effective. The number of HIV-positive patients combining antiretroviral with plant-based malaria treatment in the present study was higher (9.2%) than in Chukwuocha et al., in Nigeria (3.5%) [42]. According to Chukwuocha et al., the adjusted odds of not stopping ART were 6.14 (95% CI: 1.73–21.80) for patients who took herbs to treat their malaria compared to others taking generic medicines [42]. Other studies in sub-Saharan Africa have found that HIV-infected patients use herbal treatment while on antiretroviral therapy for seemingly minor conditions (including malaria) in the belief that it increases their energy and immunity [18]. Although the number of people treating their malaria with herbs was low in the study population and consistent with the numbers reported by [18] among HIV-positive patients on antiretroviral therapy, this finding suggests that there is a potential and underestimated risk of interaction between traditional medication and antiretroviral therapy. Studies in herbal medicines show that genetic variations in drug absorption, distribution, metabolism, and excretion, both within and between populations, can influence drug pharmacokinetics and pharmacodynamics [43, 44]. These observations would lead to treatment outcomes, drug resistance, and increased toxicity [45].

Malaria infection and viral loads showed no significant difference. However, patients with undetectable viral loads were more infected. This would be due to the fact that malaria transmission in the western region is perennial on the one hand, and on the other hand, the study took place during the rainy season when malaria transmission is high. In addition, patients with undetectable viral loads were more represented. The study conducted in West Cameroon by Tchuinkam et al. showed that malaria infection is perennial, but seasonal upstream, with malaria episodes occurring from May to August, probably due to the abundance of puddles and the attenuation of climatic constraints (temperature and relative humidity) on mosquito reproduction and survival during the previous rainy season [24]. When infectious reservoirs are provided, there could be a rapid spread of malaria into the upland bangs from the lowlands [23]. Work has shown that people living in malaria-endemic areas who are considered semi-immune to malaria are still more likely to develop clinical malaria if they are infected with HIV [9]. Malaria is endemic in Cameroon, which would predispose HIV-positive patients with a viral load of less than 50 copies per mL of blood.

The majority of participants had a parasite density above 400 trophozoites/*μ*L. However, our results do not corroborate those conducted in Bamenda [26]. They observed that patients with a parasite density of less than 200 trophozoites/*μ*L of blood were more numerous among the infected. They observed that patients with trophozoites less than 200 trophozoites/*μ*L of blood were more numerous among those infected. This difference could be attributed to a regular absence of cotrimoxazole as prophylaxis by the latter. Indeed, the active ingredients of cotrimoxazole (trimethoprim + sulfamethoxazole) could have some therapeutic effects against malaria parasites, as demonstrated by some studies [46, 47].

The prevalence of malaria by age, sex, marital status, and education level did not vary significantly, suggesting that they use effective malaria control methods such as sleeping under impregnated mosquito nets and cleaning the environment and stagnant water.

Information collected from 585 patients through a questionnaire shows that 81.9% knew that malaria is dangerous. This high rate of knowledge is thought to be due to media coverage, sensitization during surveys, or by staff at the approved HIV treatment center.

The largest number of patients associated malaria transmission with mosquito bites. This result is consistent with that recorded by Tay et al. in Techiman, Ghana [48]. It showed that 91.2% of the respondents knew the mode of transmission of malaria. The high level of knowledge of the participants on the mode of transmission of malaria would be related to the fact that they benefit from recurrent awareness campaigns conducted by malaria control programs. It should be noted that the level of knowledge of the populations regarding the mode of transmission of a disease is an indicator to guide efficient strategies for the control of that disease [49]. However, few patients have knowledge about the type of mosquito responsible for disease transmission (10.4%). During health campaigns and other awareness activities, the focus is on the pathogen.

The distribution of the number of malaria attacks per year shows that 67.2% of participants reported having at least one malaria attack per year. This result is lower than that of Chukwuocha et al., where they found 95% [42]. This is due to the fact that Nigeria has the largest malaria burden in the central African subregion [6].

In terms of net use, 69.9% slept under LLINs. This high rate could be explained by patients' fear of being infected with malaria. This result is lower than the target recommended by the WHO (80%) [50]. In contrast, the study in Western Kenya showed that 84.3% of HIV-positive patients slept under bed nets [51], and in Cameroon, 57.4% of people are living with HIV in Buea [52]. This practice can significantly reduce the rate of malaria. According to Atieli et al. [53] in Kenya, sleeping under the treated nets significantly reduces the risk of human-vector contact and, consequently, malaria infection.

The average amount spent on malaria treatment during the last crisis was 7364.1 CFA francs (€11.24). This is almost one-third of the guaranteed minimum wage in Cameroon. The majority of the population had one to three malaria attacks per year. These are a low-income group. Because of the number of malaria attacks per year and their low income, it is necessary to take a positive look at the implementation of surveillance of these people living with HIV.

The level of knowledge about the signs and symptoms of malaria reported in the study was high. This may be due to the fact that people living in malaria-endemic areas are more likely to be better informed about the disease and undergo regular check-ups.

## 5. Conclusion

The present study conducted at the Bafoussam Regional Hospital consisted of identifying the species and prevalence of malaria with associated factors in HIV+ patients, followed and eligible for viral load. *Plasmodium vivax* is another species associated with *Plasmodium falciparum* that is permanently targeted in the Western region and can infect HIV-positive patients on antiretroviral therapy. The low prevalence of malaria in the present study shows that adherence to HIV antiretroviral therapy reduces the risk of malaria, as does the density of *Plasmodium* spp. as a secondary function of antiretrovirals. Therefore, HIV+ patients should be diagnosed and treated regularly in order to reduce the prevalence of malaria and cases of recrudescence.

## Figures and Tables

**Table 1:** Sociodemographic characteristics of people living with HIV at the Bafoussam Regional Hospital, Cameroon in 2021; age and level of education of HIV-positive patients according to gender.

**Parameter**	**Category**	**Frequency**	**Percentage**	**Gender**	
				**Female ** **n** ** (%)**	**Male ** **n** ** (%)**
Gender	Female	431	73.67		
	Male	154	26.32		

Age group (years)	≤ 20	33	5.64	19 (57.6)	14 (42.4)
	21–30	67	11.45	64 (95.5)	3 (4.5)
	31–40	124	21.19	100 (80.6)	24 (19.4)
	41–50	178	30.42	133 (74.7)	45 (25.3)
	51–60	132	22.56	86 (65.2)	46 (34.8)
	> 60	51	8.71	29 (56.9)	22 (43.1)

Marital status	Single	135	23.07		
	Divorced	32	5,47		
	Married	310	53		
	Widow/widower	108	18.46		

Level of education	Analphabetic	51	8.71	45 (88.2)	6 (11.8)
	Primary	214	36.58	164 (76.6)	50 (23.4)
	Secondary	275	47	197 (71.6)	78 (28.4)
	University	45	7.69	25 (55.6)	22 (43.1)

**Table 2:** Patients' level of education according to age.

**Parameter**	**Category**	**Age group (years)**					
		**≤ 20**	**21–30**	**31–40**	**41–50**	**51–60**	**> 60**
Level of education	Analphabetic	0 (0)	3 (5.9)	6 (11.8)	14 (27.5)	15 (29.4)	13 (25.5)
	Primary	8 (3.7)	20 (9.3)	48 (22.4)	61 (28.5)	55 (25.7)	22 (10.3)
	Secondary	25 (9.1)	37 (13.5)	58 (21.1)	89 (32.4)	52 (18.9)	14 (5.1)
	University	0 (0)	7 (11.5)	12 (26.7)	14 (31.1)	10 (22.2)	2 (4.4)

**Table 3:** *Plasmodium* species and density according to sex and age.

**Parameter**	**Category**	***P. v*** **n** ** (%)**	***P. f*** **n** ** (%)**	**84–400 ** **n** ** (%)**	**401–8260 ** **n** ** (%)**
Gender	Female	2 (6.3)	30 (93.8)	12 (37.5)	30 (93.8)
	Male	0 (0)	14 (100)	5 (35.7)	9 (64.3)

Age group (years)	≤ 20	1 (33.3)	2 (66.7)	0 (0)	3 (100)
	21-30	0 (0)	4 (100)	2 (50)	2 (50)
	31-40	0 (0)	12 (100)	5 (33.3)	8 (66.7)
	41-50	0 (0)	10 (100)	5 (50)	5 (50)
	51-60	1 (8.3)	11 (91.7)	4 (33.3)	8 (66.7)
	> 60	0 (0)	5 (100)	2 (40)	3 (60)
	Total	2 (4.3)	44 (95.7)		

*Note:n* (%) = number of species (percentage).

Abbreviations: *P. f =  Plasmodium falciparum, P. v = Plasmodium vivax.*

**Table 4:** Prevalence of malaria parasites according to division.

**Category**	**Total ** **N** ** (%)**	**Infected ** **n** ** (%)**	**OR (95% IC)**	**p** ** value**
*Division*				
Bamboutos	47 (8)	2 (4.3)	1^a^	
Haut-Plateaux	21 (3.6)	1 (4.8)	1.12 (0.09–13.13)	0.92
Mifi	366 (63)	25 (6.8)	1.64 (0.37–7.19)	0.5
Menoua	17 (3)	1 (5.9)	1.4 (0.11–16.58)	0.78
Haut-Nkam	3 (0.5)	1 (33.3)	11.25 (0.69–182)	0.08
Koung-Khi	20 (3.4)	1 (5.0)	1.18 (0.10–13.85)	0.89
Nde	11 (2)	2 (18.2)	5 (0.62–40.28)	0.13
Noun	63 (11)	7 (11.1)	2.81 (0.55–14.2)	0.21
Others	37 (6.3)	6 (16.2)	4.35 (0.82–23)	0.08

*Note:* Superscripted values with the same letters are not significantly different at *p* = 0.05. *n* (%) = number of individuals (percentage).

Abbreviation: OR (95% CI) = odds ratio (95% confidence interval).

^a^Reference category.

**Table 5:** Malaria prevalence according to clinical factors and monthly income.

**Parameter**	**Variable**	**Total ** **N**	**Infected ** **n** ** (%)**	**OR (95% IC)**	**p** ** value**
Duration on ARV	≤ 36 months	91	11 (12.1)	1.8 (0.87–3.7)	0.1
	> 36 months	494	35 (7.1)	1	

Type of treatment	1^st^ line	541	44 (8.1)	0.8 (0.04–15.19)	0.88
	2^nd^ line	40	2 (5.0)	0.58 (0.02–14.18)	0.74
	3^rd^ line	4	0 (0)	1	

Monthly income in CFA francs	< 35,000	398	30 (7.5)	1	
	≥ 35,000	187	16 (8.6)	1.1 (0.6–2.17)	0.7

Abbreviation: ARV = antiretroviral.

**Table 6:** Prevalence of malaria according to sociodemographic factors.

**Parameter**	**Category**	**Total ** **N** ** (%)**	**Infected ** **n** ** (%)**	**OR (95% IC)**	**p** ** value**
Gender	Female	431	32 (7.4)	1^a^	
	Male	154	14 (9.1)	1.24 (0.64–2.40)	0.51

Age group (years)	≤ 20	33	3 (9.1)	1.68 (0.43–6.46)	0.45
	21–30	67	4 (6.0)	0.80 (0.21–3.00)	0.74
	31–40	124	12 (9.7)	1.80 (0.70–4.01)	0.18
	41–50	178	10 (5.6)	1^a^	
	51–60	132	12 (9.1)	1.68 (0.70–4.01)	0.24
	> 60	51	5 (9.8)	1.82 (0.59–5.60)	0.29

Marital status	Single	135	14 (10.4)	1.96 (0.72–5.30)	0.18
	Divorced	32	2 (6.3)	1.13 (0.21–5.90)	0.88
	Married	310	24 (7.7)	1.42 (0.56–3.58)	0.45
	Widow/widower	108	6 (5.7)	1^a^	

Level of education	Analphabetic	51	6 (11.8)	2.86 (0.54–14.98)	0.21
	Primary	214	14 (6.5)	1.50 (0.32–6.86)	0.59
	Secondary	275	24 (8.7)	2.05 (0.46–9.01)	0.33
	University	45	2 (4.4)	1^a^	

^a^Reference category.

**Table 7:** Prevalence of malaria according to the knowledge, attitudes, and practices towards malaria of patients living with HIV.

**Parameter**	**Category**	**Total ** **N** ** (%)**	**Infected ** **n** ** (%)**	**OR (95% IC)**	**p** ** value**
Malaria is dangerous?	Yes	479	40 (8.4)	2.1 (0.62–6.9)	0.24
	No	71	3 (4.2)	1^a^	
	Do not know	35	3 (8.6)	2.13 (0.41–11.12)	0.37

Means of transmission	Mosquito bites	434	37 (8.5)	0.44 (0.01–7.10)	0.07
	Fly/insect bites	24	2 (8.3)	0.01 (0.01–10.58)	0.53
	Lack of cleanliness	3	0 (0.0)	0.43 (0.01–33.60)	0.70
	Through food	1	0 (0.0)	1^a^	
	Do not know	123	7 (5.7)	0.19 (0.01–5.16)	0.33

NGMCM	Yes	61	3 (4.9)	1^a^	
	No	524	43 (8.2)	1.73 (0.52–5.75)	0.37

Sleeping under an LLIN	Yes	26	3 (11.5)	1.56 (0.45–5.42)	0.45
	No	559	43 (7.7)	1^a^	

Provenance of LLINs	Ministry of Public Health	456	40 (8.8)	0.49 (0.02–10.3)	0.64
	Prenatal visit	55	3 (5.5)	0.33 (0.01–8.37)	0.50
	Buy	7	0 (0.0)	0.33 (0.01–21.64)	0.6
	Other means	2	0 (0.0)	1^a^	
	Absent	65	3 (4.6)	0.28 (0.01–7.01)	0.44

*Note:n* (%) = number of individuals (percentage).

Abbreviation: NGMCM = name of the genus of mosquito that causes malaria.

^a^Reference category.

**Table 8:** Knowledge attitudes and practices towards malaria of patients living with HIV.

**Variable**	**Frequency ** **n** ** (%)**
*Is malaria dangerous?*	
Yes	479 (81.9)
No	71 (12.1)
Do not know	35 (6)
*Means of transmission*	
Mosquito bites	432 (74.2)
Fly/insect bites	24 (4.1)
Lack of cleanliness	3 (0.5)
Through food	1 (0.2)
Do not know	123 (21)
*Name of the genus of mosquito that causes malaria*	
Yes	61 (10.4)
No	524 (89.6)
*Sleeping under an LLIN*	
Yes	409 (69.9)
No	176 (30.1)
*Provenance of LLINs*	
Ministry of Public Health	456 (77.9)
Prenatal visit	55 (9.4)
Buy	7 (1.2)
Other means	2 (0.3)
Absent	65 (11.1)
*Number of crisis per year*	
Zero times	192 (32.8)
One time	189 (32.3)
Two times	126 (21.5)
Three times	51 (8.7)
Four times	15 (2.6)
Five times	12 (2.1)

## Data Availability

The data generated and analyzed during the current study are accessible but can be obtained from the corresponding author on reasonable request.
